# Visceral Artery Aneurysms in Pregnancy and Women of Childbearing Age: A Primary and Emergency Care Approach

**DOI:** 10.3390/medicina62040716

**Published:** 2026-04-09

**Authors:** Joseph Kilby, Kay Hon, Enis D. Kocak, Cassandra Hidajat, Aaron Tran, Jacob Gordon, Chrisdan Gan

**Affiliations:** 1 Department of Vascular Surgery, Barwon Health, Geelong, VIC 3220, Australia; 2 Department of Vascular Surgery, Royal Melbourne Hospital, Melbourne, VIC 3050, Australia; 3 Faculty of Health and Medical Sciences, The University of Adelaide, Adelaide, SA 5005, Australia; 4 Kidney Transplant & Vascular Surgery, Alfred Health, Melbourne, VIC 3004, Australia; 5 Department of Vascular Surgery, Peninsula Health, Melbourne, VIC 3199, Australia; 6 Eastern Health Clinical School, Monash University, Melbourne, VIC 3004, Australia

**Keywords:** aneurysm, visceral artery, pregnancy, female, primary care, endovascular

## Abstract

*Background and Objectives*: Visceral artery aneurysms (VAAs) are rare but potentially catastrophic vascular abnormalities, particularly in pregnant patients or women of childbearing age. Rupture is often fatal for both mother and fetus, with mortality rates exceeding 70% in some series. While most VAAs are found incidentally, a subset may present acutely with nonspecific abdominal or flank pain, making early recognition and appropriate referral essential. This review article aims to provide General Practitioners (GPs) and emergency department (ED) clinicians with a practical approach to the recognition, investigation, initial management, and escalation pathways for VAAs. *Results*: Physiological and hormonal adaptations in pregnancy heighten aneurysm rupture risk. Despite this, imaging is frequently delayed. Computed tomography angiography (CTA) remains the gold standard for diagnosis and is safe in pregnancy when clinically justified, with fetal radiation exposure well below teratogenic thresholds. Guidelines from major vascular societies uniformly recommend repairing VAAs in pregnancy or women planning pregnancy irrespective of aneurysm size, and treating pseudoaneurysms urgently in all patients. Endovascular intervention is first-line where anatomy permits, while open or hybrid approaches remain essential in unstable presentations. The manuscript outlines practical steps for ED and GP settings, including haemodynamic stabilization, early obstetric involvement, transfer considerations for rural environments, reproductive counselling, and post-repair surveillance. *Conclusions*: With an increasing number of abdominal scans being performed in primary and tertiary settings, there is an associated increased volume of incidental findings that require work-up. This article outlines a practical investigation and management strategy for clinicians presented with VAAs, including in high-risk cohorts, emphasizing early imaging, inter-specialty coordination, and guideline-supported thresholds for intervention.

## 1. Introduction

Visceral artery aneurysms (VAAs) are defined as abnormal dilatations of the arteries supplying the abdominal organs, which exceed 1.5 times the normal vessel diameter [1]. Aneurysms may be classified as true aneurysms, involving all three arterial wall layers, or pseudoaneurysms which develop from a breach in the arterial wall caused by trauma, infection, inflammation, or iatrogenic injury [1,2]. For women in pregnancy, and during of childbearing age, true aneurysms are the predominant presentation [2,3,4].

Although rare in the general population—with an estimated prevalence of 0.1–2% based on autopsy and imaging studies—VAAs carry a disproportionate risk when rupture occurs, particularly in pregnant or multiparous women [1,5]. The splenic artery accounts for the majority (up to 60%) of VAAs, followed by the hepatic, superior mesenteric, coeliac, and renal arteries [1]. Ruptured splenic artery aneurysms accounted for 2% (9 of 449 cases) of all maternal deaths in Australia between 1997 and 2017 [6].

### Why Pregnancy Matters

**Figure 1:**
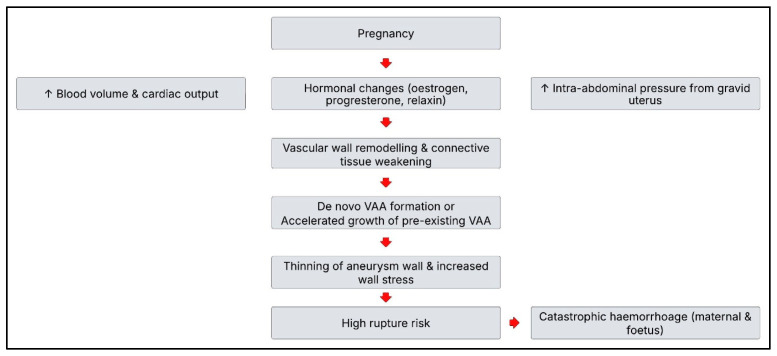
Pathophysiological mechanisms contributing to VAA rupture risk in pregnancy.

In pregnancy, several physiological and mechanical changes contribute to aneurysm growth and rupture risk as shown in Figure 1: increased blood volume and cardiac output, hormone-mediated connective tissue effects (via estrogen, progesterone, and relaxin) that weaken the arterial wall, uterine enlargement and mechanical displacement of abdominal organs and vessels, as well as hyperdynamic circulation, particularly in the third trimester [5,7].

These changes may lead to de novo aneurysm formation or accelerated growth of previously stable aneurysms [5]. Rupture in pregnancy is associated with maternal mortality approaching 70% and fetal mortality up to 90%, making early detection and pre-emptive treatment essential [7].

Despite these risks, most VAAs are diagnosed either incidentally or after rupture [5,7]. Presentation is often vague and easily mistaken for more common conditions such as renal colic, ectopic pregnancy, placental abruption, or gastrointestinal perforation. Compounding the issue, clinicians may delay imaging due to concerns about fetal radiation exposure [3,4,8].

The role of general practitioners and emergency clinicians lies in maintaining a high index of suspicion, initiating timely imaging, and escalating care promptly. International guidelines now recommend intervention irrespective of aneurysm size in pregnancy and women of childbearing potential, or in cases of pseudoaneurysm or active symptoms [3,4,8,9].

## 2. Investigation Strategy

For clinicians in primary or emergency care settings, early imaging plays a pivotal role in the diagnosis and risk stratification of VAAs [1,3,10]. While concerns about fetal and young female radiation exposure are common, they should not delay appropriate investigation [4,7].

Computed tomography angiography (CTA) remains the gold standard for anatomical assessment [1]. Modern multi-slice scanners, when used with appropriate shielding and protocols, expose the fetus to radiation levels well below the threshold for deterministic effects [4]. Magnetic resonance angiography (MRA) offers an alternative in patients with contraindications to iodinated contrast, although its use in acute scenarios is limited by availability and longer acquisition times [10]. In the absence of conclusive long-term data on its effects on the fetus, gadolinium-enhanced MRA can be used in situations where the benefit (e.g., investigation of a life-threatening aneurysm) clearly outweighs this risk [11,12]. For bedside triage, Doppler ultrasound may identify larger aneurysms or flow abnormalities but is limited by operator skill, body habitus and the gravid uterus [1]. It also does not provide the anatomic detail required to plan intervention in this high-risk patient group.

Importantly, fetal exposure from a single abdominal CTA is typically <25 mGy, whereas teratogenic thresholds lie above 100 mGy [4,12]. Shielding the fetus offers benefit at all stages of pregnancy. In the first trimester, the risk of radiation-induced deterministic effects is greatest due to organogenesis and the heightened radiosensitivity of developing tissues. In the second and third trimesters, shielding remains important as fetal tissues remain susceptible to scatter radiation.

In all trimesters, the risk of a missed or delayed diagnosis outweighs the low radiation burden of a single scan [3,4]. While most VAAs are asymptomatic, the presence of sudden severe abdominal pain, haemodynamic instability, syncope, anemia, or fetal bradycardia on cardiotocography should raise a high index of suspicion for rupture, prompting urgent CTA [5]. GPs and ED clinicians should feel confident to initiate imaging based on clinical suspicion and promptly involve radiology and vascular surgery teams to assist with next steps.

## 3. Management Principles

Once a VAA is identified, clinical stability and aneurysm characteristics guide initial decision-making [1,2,3,9]. Hemodynamically unstable patients require urgent resuscitation and intervention. Stable patients can undergo more detailed imaging and elective repair. Regardless of acuity, multidisciplinary evaluation, management, and follow-up is indicated [3,9].

Endovascular treatment has become first-line for anatomically suitable aneurysms [3,4,8,9]. Coil embolization and covered stent placement are commonly employed, with good outcomes and lower procedural morbidity compared to open surgery [13,14,15]. Stent-assisted embolization may also be used in instances of wide-neck or geometrically complex aneurysms [8]. Open surgical repair remains essential in unstable patients, in anatomically complex aneurysms, or where endovascular access is not feasible [2,3]. Examples of open surgical repair include primary repair (aneurysmorraphy), resection and re-anastomosis or bypass, ligation and sacrifice (e.g., splenic or distal renal artery). Hybrid approaches combining endovascular and open surgical components may be employed selectively [2]. In the emergent setting, aneurysm intervention is often performed concurrently with obstetric delivery in a hybrid operating suite.

Guidelines from multiple vascular societies highlight pregnancy, pseudoaneurysm, and symptomatic aneurysm as indications for repair regardless of size (Table 1) [3,4,8,9]. In women of childbearing potential, the decision to intervene is more nuanced and often based on operative risk and reproductive planning [3,9]. However, guidelines generally recommend considering elective treatment in this patient cohort, irrespective of size, due to the exceptionally high maternal and fetal mortality rates associated with rupture and the relative safety of elective intervention in stable settings [3,5,7].

In the absence of specific guidelines for visceral artery aneurysms, emergent assessment and care is guided by the principles underpinning abdominal aortic aneurysm management [15,16,17]:

• Hemodynamic assessment,
• Intravenous access and resuscitation guided by a permissive hypotension strategy,
• Immediate referral to vascular surgery and/or interventional radiology,
• Imaging if stable and not yet performed, and
• Early contact with obstetrics if pregnancy is confirmed or suspected.

Timely communication and early escalation are critical to preventing rupture and facilitating definitive management in a tertiary setting. Until the patient has been reviewed by a vascular surgeon, and undergone exclusion of their aneurysm, they should be closely monitored and offered contraceptive counselling if not yet pregnant.

## 4. Other Special Considerations

Pseudoaneurysms represent a distinct and more dangerous clinical entity, with a substantially higher risk of rupture than true aneurysms [1,2,3]. This is reflected in mortality rates approaching 90% if untreated [18]. Accordingly, pseudoaneurysms warrant urgent treatment irrespective of size, particularly when associated with infection, inflammation, or prior intervention [1,2,3]. The splenic artery is most frequently involved, followed by the hepatic artery [18]. Hepatic artery pseudoaneurysms typically occur after blunt hepatic trauma or iatrogenic injury and commonly present with gastrointestinal bleeding or haemobilia [3]. Splenic artery pseudoaneurysms are most often associated with pancreatitis, although non-iatrogenic blunt trauma is increasingly recognized [18].

Visceral artery aneurysms are heterogenous in their anatomical distribution, etiology, rupture risk, and management [3,4,9,19]. While general principles of early investigation and urgent management remain consistent (Table 1), important vessel-specific differences exist in rupture-associated mortality, pregnancy-related risk, and thresholds for intervention. Table 2 summarizes these artery-specific characteristics to provide a comparative overview of current evidence and management strategies.

Clinical decision-making should also account for local resource availability. In rural or resource-limited settings, the threshold for transfer should be low [1,3]. GPs and ED physicians practicing in such environments should be empowered to initiate early referral to tertiary centres with vascular surgery and interventional radiology services [1,3]. Delays in accessing definitive care may increase the risk of rupture and limit treatment options [5,7].

Multidisciplinary coordination is essential and should include vascular surgeons, interventional radiologists, and obstetricians [2,3,9]. Early involvement of these teams allows for tailored planning and optimization of maternal and fetal outcomes, particularly if repair is undertaken during pregnancy [3,5,7].

### 4.1. Follow-Up and Future Reproductive Counselling

Following successful aneurysm treatment, ongoing surveillance and careful reproductive planning are essential, given the risk of aneurysm progression or rupture in subsequent pregnancy [3,9,10]. While recurrence rates are low, follow-up imaging is recommended to assess for residual aneurysm sac expansion, repair patency, and progression at other visceral sites [1,10]. Surveillance should be individualized based on intervention type and patient-specific risk factors, typically incorporating CTA as the initial post-operative modality to confirm aneurysm exclusion and detect complications, including endoleak. Subsequent follow-up may transition to duplex ultrasound to minimize radiation exposure, with CTA reserved where sonographic assessment is inadequate. Imaging is commonly performed at 6- to 12-month intervals [1,10]. Given the potential for late complications and disease progression, lifelong surveillance is recommended, with ongoing follow-up under the care of the treating vascular surgeon or interventionalist.

Women of childbearing age should be counselled regarding the implications of VAA repair on future pregnancies [3,5,9]. Although post-treatment pregnancy is generally considered safe following successful aneurysm exclusion, there is limited prospective data [1,5]. In such cases, a pre-conception vascular review and multidisciplinary birth planning is advisable, particularly for women who underwent complex or emergency repair [5,7].

Importantly, the presence of a VAA may represent an underlying arteriopathy or connective tissue disorder. If additional vascular anomalies are found or the patient is young with no apparent risk factors, referral for genetic evaluation or specialist input is warranted [1,19].

Additionally, clinicians should consider the organ-specific implications of treatment. For example, in cases where splenic artery embolization compromises splenic function, long-term management may require vaccination and prophylaxis against encapsulated organisms.

In all patients, post-treatment care should include patient education, cardiovascular risk factor modification, and documentation to alert future providers of the aneurysm history and treatment pathway [1,3].

### 4.2. Future Directions and Recommendations

Despite increasing recognition of visceral artery aneurysms in pregnancy and women of childbearing age, significant knowledge gaps remain. Much of the current evidence is derived from retrospective studies, case series, and registry data, reflecting the rarity of these conditions. Consequently, the natural history of small asymptomatic aneurysms in women of reproductive age remains poorly defined, and optimal thresholds for intervention and surveillance continue to be debated.

Further research should focus on better characterizing pregnancy-specific risk factors for aneurysm formation and rupture, including the role of hormonal influences, haemodynamic changes, and underlying arteriopathies. The potential value of targeted screening strategies in high-risk populations, such as multiparous women or patients with connective tissue disorders, also warrants investigation.

Although advances in endovascular techniques have expanded treatment options, prospective data evaluating maternal and fetal outcomes following intervention during pregnancy remain limited. Multicentre registries and collaborative studies would facilitate more robust evaluation of treatment strategies, long-term outcomes, and the safety of imaging and contrast exposure in this patient population.

Future efforts should prioritize development of pregnancy-specific management pathways, improved risk stratification tools, and standardized surveillance protocols. International collaboration between vascular, obstetric, and radiological societies may help establish consensus guidance and optimize maternal and fetal outcomes while minimising unnecessary intervention.

## 5. Conclusions

Visceral artery aneurysms, although rare, represent a high-risk pathology in pregnancy and women of childbearing age, with rupture associated with significant maternal and fetal mortality. Early diagnosis, supported by appropriate imaging, and prompt involvement of vascular and obstetric teams are critical to optimize outcomes. Clinicians in primary and emergency care settings play a key recognizing potential presentations and facilitating timely referral. Current guidelines support a proactive approach to management, with intervention recommended irrespective of aneurysm size in high-risk cohorts. While endovascular techniques have expanded treatment options, open and hybrid approaches remain critical in unstable or anatomically complex cases. Long-term surveillance and reproductive counselling are integral to care, given the potential for disease progression and future pregnancy-related risk.

## 6. Key Points for Practice

• VAAs are rare but carry a high risk of rupture in pregnancy. Rupture is associated with significant maternal and fetal mortality rates [5,7].
• A high index of suspicion should be maintained in pregnant or postpartum women with unexplained abdominal or flank pain [5,7].
• Imaging with CTA is safe in pregnancy when clinically indicated—early diagnosis can be lifesaving [3,4,8].
• International guidelines recommend treatment of VAAs in pregnancy or women of childbearing age regardless of size [3,4,8,9].
• Endovascular repair is first-line for suitable stable aneurysms. Open surgical management remains essential in unstable or complex cases [2,6,10].
• Early involvement of vascular surgery, radiology, and obstetrics is critical for optimal maternal and fetal outcomes [3,5,9].
• Post-repair counselling should include reproductive planning and aneurysm surveillance [3,9,10].

## Figures and Tables

**Table 1:** Recommended intervention thresholds for VAAs in pregnancy and women of childbearing age.

Patient Scenario	Intervention Threshold	Supporting Guidelines/Evidence
Pregnant (any trimester)	Repair all true aneurysms regardless of size	SVS 2020 [3], ESVS 2025 [4], Aung et al., 2023 [5] CIRSE 2024 [8].
Women of childbearing potential	Repair all true aneurysms regardless of size if operative risk is acceptable	SVS 2020 [3], ESVS 2025 [4], Pratesi 2023 [9].
Planning pregnancy (not yet pregnant)	Repair all true aneurysms regardless of size	CIRSE 2024 [8], Pratesi 2023 [9].
Pseudoaneurysm (any patient)	Repair regardless of size or symptoms	CIRSE 2024 [8], Pratesi 2023 [9], Obara 2019 [15]

**Table 2:** Overview of common visceral artery aneurysms, relationship with pregnancy, mortality on rupture, and criteria for repair.

Visceral Artery	Etiology and Relationship with Pregnancy	Indications for Treatment	Surveillance
**Splenic artery**	Associated with 20–50% of all ruptures [3]. 80% maternal and 90% fetal mortality rates [3]. Almost exclusively rupture in the third trimester [19]. Most common VAAs, comprising 60% of all VAAs [19]. Multiparity is a risk factor for development of SMA aneurysms [19].	Rupture [3]. Asymptomatic, non-ruptured aneurysm of any size in women of childbearing age [3,4]. Asymptomatic, non-ruptured aneurysm of any size in pregnant patient [3,4]. Treatment of non-ruptured pseudoaneurysms of any size [3]. Asymptomatic aneurysm ≥30 mm [4].	Annual CT or DUS [3]. Periodic surveillance post endovascular intervention to assess for endoleak and aneurysm reperfusion [3].
**Hepatic artery**	No known association with pregnancy [19]. Mortality from rupture 20–70% [19]. Pseudoaneurysms account for 25–80% of all cases, often iatrogenic or traumatic [3].	Asymptomatic aneurysm ≥30 mm [4]. Any pseudoaneurysm regardless of size should be treated on diagnosis [3]. All symptomatic aneurysms regardless of size [3,4].	Annual follow-up with CT to observe patients with asymptomatic aneurysms [3].
**Coeliac artery**	Account for approx. 4% of all VAAs (Rutherford); prevalence seen at autopsy closer to 1 in 8000 [19]. Becoming increasingly common due to improvements in imaging [3]. Rupture rates 10–20%, mortality post-rupture approaches 50% [19].	Treatment should be considered for coeliac artery aneurysms >30 mm [4]. or >20 mm [9]. Treatment should be considered for all coeliac artery pseudoaneurysms [9]. Treatment should be considered in pregnant women/childbearing age regardless of size [9].	Surveillance should be considered for patients with asymptomatic coeliac artery aneurysms <30 mm [4]. Periodic surveillance should be considered for endovascular treatment to assess for endoleak or ongoing aneurysm perfusion [3].
**Superior mesenteric artery**	Represent 3.5–8% of all VAAs [19]. Non-mycotic etiology most common, compromising 92% [4].	Treatment of all SMAAs with endovascular-first approach [3]. Endovascular or open treatment considered for SMAAs >35 mm [4]. Treatment of all mycotic or symptomatic SMAAs [4].	Surveillance should be considered for patients with asymptomatic SMAAs <30 mm [4].
**Renal artery**	Pregnancy associated with increased rupture [4]. 5.1% maternal mortality rate is estimated, fetal mortality however is 38% [4] but up to 85% [3]. Increased rupture in pregnancy thought to be due to mass effect from gravid uterus, hormonal changes to the vessels, increased vascular volume and flow [3].	Treatment of aneurysms of any size in pregnancy [4]. Asymptomatic aneurysms should be treated if ≥30 mm [4]. Asymptomatic patients of childbearing age should have treatment regardless of size [3].	Annual surveillance should be considered for patients with asymptomatic aneurysms <30 mm [4]. Annual surveillance of non-operative patients until 2 consecutive studies demonstrate stability, then can go to 2–3 yrly studies [3]. Patients with renal artery aneurysms should be screened for fibromuscular dysplasia [3].

## Data Availability

No new data were created.

## Abbreviations

The following abbreviations are used in this manuscript:

VAAs	Visceral artery aneurysms
GPs	General Practitioners
CTA	Computed tomography angiography
MRA	Magnetic resonance angiography
ED	Emergency department
SVS	Society for Vascular Surgery
ESVS	European Society of Vascular Surgery
CIRSE	Cardiovascular and Interventional Radiological Society of Europe
